# Lycopene, Carotenoids, and Retinoids in Cancer Chemoprevention: Molecular Mechanisms and Clinical Implications

**DOI:** 10.3390/nu18142318

**Published:** 2026-07-15

**Authors:** Ecem Kalemoglu, Kazim Sahin, Nurhan Sahin, Omer Kucuk

**Affiliations:** 1 Department of Medicine, Rutgers University-Jersey City Medical Center, Jersey City, NJ 07302, USA; ecem.kalemoglu@rutgers.edu; 2 Department of Animal Nutrition, Faculty of Veterinary Science, Firat University, 23119 Elazig, Turkey; nsahinkm@yahoo.com (K.S.);; 3 Winship Cancer Institute of Emory University, Atlanta, GA 30322, USA; 4 Department of Urology, Emory University School of Medicine, Atlanta, GA 30322, USA; 5 Department of Hematology and Medical Oncology, Emory University School of Medicine, Atlanta, GA 30322, USA

**Keywords:** carotenoids, lycopene, β-carotene, α-carotene, retinoids, retinoic acid, chemoprevention, antioxidants, oxidative stress, inflammation

## Abstract

Cancer development arises from dynamic interactions between inherited susceptibility and modifiable environmental exposures, among which diet plays a central role. Carotenoids, lipophilic plant-derived pigments including lycopene, α-carotene, and β-carotene, and retinoids, the vitamin A derivatives that regulate gene transcription via retinoic acid receptors (RARs) and retinoid X receptors (RXRs), have been extensively investigated for their chemopreventive and therapeutic potential. This review aims to provide an integrated, mechanism-based synthesis of the roles of lycopene, α- and β-carotene, and retinoids in cancer chemoprevention and to clarify the conditions under which they are most likely to be effective. Beyond summarizing established antioxidant and nuclear-receptor mechanisms, we highlight as a novel emphasis the epigenetic actions of these compounds, including effects on DNA methylation, histone modification, and microRNA regulation, and we integrate these with the well-recognized divergence between dietary and high-dose supplement outcomes. Experimental evidence demonstrates that carotenoids modulate oxidative stress, inflammation, proliferation, apoptosis, angiogenesis, and metastasis through pathways such as Nrf2/ARE, NF-κB, STAT3, Akt/mTOR, MAPK, and Wnt/β-catenin. Lycopene, in particular, exhibits strong antioxidant capacity and multi-target signaling effects, while provitamin A carotenoids additionally influence retinoid-mediated transcriptional programs. Retinoids exert broader differentiation-inducing and antiproliferative effects through direct nuclear receptor signaling and represent one of the few successful differentiation therapies in oncology, most notably in acute promyelocytic leukemia. Epidemiologic studies generally associate higher dietary carotenoid intake with reduced risk of several malignancies, including prostate, breast, lung, colorectal, and gastric cancers. However, randomized trials of isolated high-dose supplementation, particularly β-carotene in smokers, have demonstrated null or harmful effects, highlighting a critical divergence between whole-food dietary patterns and pharmacologic supplementation. In conclusion, carotenoids and retinoids possess biologically plausible anticancer properties, yet their clinical utility remains context dependent. Future research should prioritize biomarker-guided, precision-based strategies, standardized formulations, and whole-food dietary approaches to clarify their role in cancer prevention and treatment.

## 1. Introduction

Cancer is a major global health burden influenced by both genetic and environmental factors, including diet. Increasing evidence indicates that dietary patterns can modulate cancer risk, particularly through effects on inflammation, oxidative stress, and immune responses. Diets high in red and processed meats and low in fruits and vegetables are associated with increased inflammatory markers linked to carcinogenesis, whereas diets rich in phytochemicals, vitamins, and minerals with antioxidant and anti-inflammatory properties are associated with reduced cancer risk [1,2]. Over the past two decades, converging epidemiological evidence has shifted the focus from single nutrients toward broader dietary patterns, demonstrating that plant-forward, anti-inflammatory dietary profiles, including Mediterranean, DASH, and Healthy Eating Index–based patterns, are consistently associated with reduced risk of lung, colorectal, pancreatic, and breast cancers [3,4,5,6,7,8]. In contrast, Western-style diets characterized by high consumption of red and processed meats, refined carbohydrates, and pro-inflammatory components are linked to elevated cancer risk and higher dietary inflammatory index scores [3,9,10]. These associations implicate chronic inflammation, oxidative stress, and immune dysregulation as central mechanisms linking diet to carcinogenesis. Within this framework, carotenoids have attracted sustained interest as candidate chemopreventive agents. Carotenoids are lipophilic, polyene pigments synthesized by plants and photosynthetic organisms and structurally classified into carotenes (α-carotene, β-carotene, lycopene) and xanthophylls (lutein, zeaxanthin, β-cryptoxanthin) [1,11]. Several of these compounds accumulate in human plasma and tissues, and higher dietary intake or circulating levels have been inversely associated with total and site-specific cancer risk across multiple meta-analyses and umbrella reviews [1,12,13,14]. Traditionally, the anticancer properties of carotenoids were attributed primarily to their antioxidant capacity, particularly their ability to quench singlet oxygen and reduce lipid peroxidation [13,15]. However, accumulating mechanistic data indicate that carotenoids function not merely as radical scavengers but as modulators of redox-sensitive transcriptional networks [13]. These include activation of the Nrf2/ARE pathway, suppression of NF-κB and STAT3 signaling, regulation of PI3K/Akt and MAPK cascades, and modulation of p53-dependent apoptosis [1,16,17]. Provitamin A carotenoids are additionally cleaved by BCO1/BCO2 to generate retinoids that engage nuclear retinoic acid receptors (RARs) and retinoid X receptors (RXRs), thereby directly influencing gene transcription programs governing differentiation, proliferation, and immune responses [13,15]. In contrast, non-provitamin A carotenoids such as lycopene exert transcriptional and signaling effects largely independent of classical retinoid receptor activation.

Despite this compelling biological rationale, clinical translation has been inconsistent. Observational studies repeatedly report inverse associations between carotenoid-rich diets and cancer risk, yet randomized controlled trials of isolated high-dose supplementation, most notably β-carotene in smokers, have demonstrated null or even harmful outcomes [12,15,18]. This divergence challenges the simplistic antioxidant paradigm and underscores the need to reconsider carotenoids within a systems biology context encompassing redox threshold effects, genetic modifiers, metabolic cleavage products, and food-matrix interactions. In this review, we re-evaluate lycopene, α- and β-carotene, and retinoids through an integrated mechanistic lens that connects redox biology, nuclear receptor signaling, and inflammatory regulation with epidemiologic and clinical trial evidence. By examining the molecular determinants of benefit versus harm, including dose, oxidative microenvironment, and host genetic context, we aim to clarify the conditions under which carotenoids and retinoids may function as effective chemopreventive agents and to define a precision-oriented framework for future research. The primary aim of this review is therefore to provide an integrated, mechanism-oriented synthesis of how lycopene, α- and β-carotene, and retinoids influence carcinogenesis, and to define the conditions under which they are most likely to be effective. Its main novel contribution is to bring together, within a single framework, mechanistic layers that are usually treated separately: redox and nuclear-receptor signaling, the dietary-versus-supplement “supplementation paradox,” and, in particular, the epigenetic actions of these compounds on DNA methylation, histone modification, and microRNA regulation, which have received little attention in previous reviews. By linking these mechanisms to epidemiologic and clinical-trial evidence, the review proposes a precision-oriented framework intended to guide biomarker-based patient selection and future trial design.

### Search Strategy and Study Selection

This narrative review was informed by a structured search of the PubMed, Scopus, and Web of Science databases, supplemented by Google Scholar and by hand-searching the reference lists of relevant articles. Searches combined the terms “lycopene,” “carotenoid,” “α-carotene,” “β-carotene,” and “retinoid” or “retinoic acid” with terms such as “cancer,” “chemoprevention,” “clinical trial,” “mechanism,” “antioxidant,” “nuclear receptor,” “epigenetic,” “DNA methylation,” “histone,” and “microRNA,” using Boolean operators. We considered peer-reviewed articles published in English through February 2026, including preclinical studies, epidemiologic studies, randomized controlled trials, and prior reviews. Priority was given to randomized trials, meta-analyses, and mechanistic studies of direct relevance to the chemopreventive activity of carotenoids and retinoids.

## 2. Chemistry and Classification of Carotenoids and Retinoids

Carotenoids are the second-most-abundant natural pigments after chlorophyll, with over 1100 identified across about 700 organisms [19]. They are lipophilic yellow, red, and orange pigments characterized by long-chain hydrocarbons with conjugated double bonds and are widely distributed in plants and photosynthetic organisms. Structurally, they are classified into hydrocarbon carotenoids (carotenes, including α-carotene, β-carotene, and lycopene) and oxygenated carotenoids (xanthophylls such as lutein, zeaxanthin, and β-cryptoxanthin) [14,20]. Carotenes are generally nonpolar and lipid-soluble, while xanthophylls are more polar and often exhibit stronger antioxidant properties due to oxygen-containing functional groups. The biological functions and physicochemical characteristics of carotenoids are strongly determined by their structural features [19]. Because animals cannot synthesize carotenoids, they must be obtained from the diet, and humans uniquely accumulate a wide range of dietary carotenoids, with six major forms (α-carotene, β-carotene, lycopene, β-cryptoxanthin, lutein, and zeaxanthin) predominating in plasma and tissues [14,21]. In this review, we focus primarily on three major carotenoids, lycopene, α-carotene, and β-carotene, alongside a dedicated discussion of retinoids, examining mechanistic evidence for anticancer activity and the clinical and epidemiological data for each major cancer type.

## 3. Lycopene

Lycopene, a non-provitamin A carotenoid abundant in tomatoes and other red fruits, has been extensively investigated for its antioxidant properties and its ability to modulate multiple molecular pathways involved in tumor initiation and progression [22]. Major dietary sources include tomatoes and processed tomato products, as well as watermelon, papaya, guava, and pink grapefruit [23,24,25] (Table 1). Lycopene occurs predominantly in the all-trans configuration in fresh foods, whereas thermal processing promotes formation of cis-isomers with improved bioavailability [26,27,28]. Structurally, it is a C40 tetraterpene composed of eight isoprene units and characterized by an extended conjugated double-bond system, which underlies its strong antioxidant activity and enables efficient interaction with reactive oxygen species (ROS) and free radicals, giving it one of the highest singlet oxygen quenching capacities among carotenoids [23,24,25,29,30,31,32]. Experimental, epidemiological, and clinical evidence suggests that lycopene may inhibit tumorigenesis by regulating oxidative stress, inflammation, cell proliferation, apoptosis, and intercellular communication [22,33].

### 3.1. Molecular Mechanisms of Lycopene in Cancer Prevention

#### 3.1.1. Antioxidant and Redox Regulation

Lycopene is among the most efficient biological singlet oxygen quenchers and a potent lipophilic antioxidant, enabling it to attenuate oxidative DNA damage, lipid peroxidation, and ROS-driven cellular injury, key upstream drivers of mutagenesis and tumor initiation [38,39]. Importantly, contemporary evidence supports a model in which lycopene’s anticancer activity extends beyond direct radical scavenging toward redox-sensitive transcriptional control. Across experimental systems, lycopene activates the Nrf2/ARE axis and modulates the Nrf2–Keap1 regulatory node, increasing the expression of phase II detoxification and cytoprotective enzymes (e.g., HO-1, NQO1, GST, GPx), thereby reinforcing cellular resilience under oxidative and electrophilic stress [40,41,42,43,44]. In vivo and human dietary interventions with tomato products or lycopene are consistently associated with improved antioxidant enzyme activity (SOD, catalase, GPx) and reduced oxidative stress biomarkers, aligning mechanistic signaling with systemic redox phenotypes [42,45,46,47,48,49].

#### 3.1.2. Regulation of Cell Proliferation and Apoptosis

Lycopene influences tumor growth by regulating cell-cycle progression, apoptosis, and key signal transduction pathways. Experimental studies demonstrate inhibition of cyclin D1 expression, induction of G0/G1 cell-cycle arrest, and suppression of IGF-1 signaling, thereby contributing to reduced mitogenic activity [50,51,52]. Mechanistically, lycopene interferes with oncogenic kinase networks, particularly PI3K/Akt/mTOR, MAPK/ERK, and Wnt/β-catenin, thereby limiting survival signaling and proliferative transcriptional programs [53,54]. This multi-node suppression is a critical “high impact” feature because it links redox stabilization to direct inhibition of canonical tumor growth hubs, rather than relying solely on antioxidant effects. In parallel with proliferation control, lycopene promotes programmed cell death by coordinating the modulation of the intrinsic apoptotic machinery. Across tumor models, lycopene shifts the Bax/Bcl-2 balance toward apoptosis, activates caspase cascades (e.g., caspase-3/8), and modulates p53-dependent stress responses, collectively lowering the apoptotic threshold of malignant cells [55,56,57]. Notably, inhibition of Akt signaling can mechanistically explain convergence between apoptosis induction and reduced therapy resistance, since Akt/mTOR blockade diminishes pro-survival buffering and metabolic adaptation [53,54,58].

#### 3.1.3. Anti-Inflammatory and Immune-Modulatory Effects

Because chronic inflammation amplifies tumor initiation and progression, lycopene’s anti-inflammatory actions represent a second mechanistic pillar. Evidence indicates suppression of NF-κB activation and reduced expression of inflammatory mediators, including TNF-α, IL-6/IL-1β, COX-2, and iNOS, with upstream dampening of stress-activated MAPKs (e.g., JNK) and pattern-recognition pathways reported in several models [59,60]. Lycopene has also been linked to reduced STAT3 signaling, a cytokine-driven oncogenic transcription factor, providing a mechanistic bridge between inflammation control and antiproliferative outcomes [39,58,61]. In addition, inhibition of 5-lipoxygenase and related eicosanoid pathways supports a multi-target anti-inflammatory profile relevant to tumor-promoting microenvironments [54].

#### 3.1.4. Intercellular Communication and Additional Targets

Lycopene improves growth control by restoring gap junctional intercellular communication, notably through upregulation of connexin-43 (Cx43), a junctional protein frequently reduced in neoplasia, thereby reinforcing contact inhibition and tissue-level homeostasis [62,63,64,65,66]. Lycopene further limits malignant dissemination by suppressing angiogenic and invasive programs, including downregulation of VEGF signaling and reduced expression/activity of MMP-2 and MMP-9, consistent with decreased tumor vascularization, extracellular matrix remodeling, and invasion in multiple tumor systems [67].

### 3.2. Lycopene in Cancer Prevention Across Tumor Types

Across tumor types, a consistent pattern emerges: robust preclinical anticancer activity of lycopene and its metabolites, contrasted by heterogeneous population-level evidence that likely reflects differences in tissue bioaccumulation, exposure measurement (dietary intake vs. plasma vs. tissue), baseline redox/inflammatory context, and confounding by overall dietary patterns [1,38,57,61,67,68]. Below, we summarize the evidence with emphasis on mechanistic plausibility and clinical consistency.

#### 3.2.1. Breast Cancer

Breast cancer risk reflects a complex interplay between genetic susceptibility and modifiable environmental exposures, notably diet, adiposity, and physical activity patterns. Among diet-related factors, carotenoids, particularly lycopene, have been repeatedly proposed as candidate chemopreventive agents based on convergent redox, inflammatory, and growth-signaling mechanisms. In vitro evidence demonstrates that lycopene suppresses proliferation and promotes cell-cycle control in breast cancer cell lines, frequently manifesting as G0/G1 arrest and reduced cyclin-dependent kinase activity, alongside pro-apoptotic reprogramming characterized by decreased Bcl-2 and increased p53 and Bax expression [69,70,71,72,73]. Beyond canonical apoptosis markers, several studies report modulation of DNA repair and tumor suppressor networks, including BRCA1/2-related expression patterns, suggesting that lycopene may influence both genomic stability and survival signaling [38,39,69,70,71,72,73].

Mechanistically, growth suppression has also been linked to attenuation of inflammatory transcription, particularly via inhibition of NF-κB signaling at concentrations argued to be within physiologically achievable ranges under dietary exposure or optimized delivery [55,58,59,61].

Animal models provide supportive translational evidence: in chemically induced mammary carcinogenesis, lycopene supplementation has been associated with reductions in tumor incidence and burden (tumor weight/volume), consistent with integrated effects on oxidative stress and apoptotic control [61,68,74,75,76]. Notably, enhanced efficacy has been observed when lycopene is combined with other bioactives, most prominently genistein, supporting a network-based chemoprevention paradigm in which multi-component interventions outperform single agents by jointly targeting redox balance, endocrine/inflammatory signaling, and proliferation programs [39,61,76].

Epidemiological evidence in humans remains inconsistent. Some case–control studies report inverse associations between lycopene or total carotenoid intake and breast cancer risk, with reductions of up to 29% observed in certain analyses [77,78,79]. However, other cohort and nested case–control studies have found no significant association between plasma lycopene levels and breast cancer risk, although inverse relationships have been observed in analyses of mammary adipose tissue lycopene levels after adjustment for confounding factors [80,81,82]. One plausible interpretation, supported by mechanistic considerations of tissue deposition, is that single plasma measurements may insufficiently capture long-term exposure relevant to breast tissue biology; accordingly, analyses of mammary adipose tissue lycopene have in some settings shown inverse relationships after adjustment for confounding, suggesting that tissue-level biomarkers may better reflect biologically meaningful exposure than circulating snapshots [68,81,82].

Lycopene has emerged as a potential adjunctive agent that enhances the efficacy of anticancer therapies in breast cancer models. Lycopene, when combined with methotrexate, enhances apoptosis and anticancer activity [83]. A combination of lycopene with paclitaxel, tamoxifen, or docetaxel has also shown greater inhibition of hormone-positive breast cancer cell proliferation than monotherapy [84,85]. In addition, lycopene, combined with other phytochemicals, has demonstrated synergistic antioxidant and antitumor effects in experimental BC models. Combinations of tocopherol, melatonin, or genistein improved antioxidant enzyme activity, reduced oxidative stress markers, modulated apoptotic signaling, and inhibited tumor growth in animal models [75,86,87]. Lycopene combined with other carotenoids and phytonutrients, as well as multi-component dietary formulations, has similarly enhanced anticancer activity [88,89,90].

#### 3.2.2. Lung Cancer

Lung cancer remains one of the leading causes of cancer-related mortality globally and is strongly driven by chronic oxidative stress, inflammation, and carcinogen exposure, particularly from tobacco smoke and environmental pollutants. Consistent with this oxidative burden, patients with lung cancer frequently exhibit lower circulating concentrations of antioxidants, including lycopene and other carotenoids [91]. Mechanistically, lycopene is proposed to counter lung tumorigenesis through coordinated regulation of redox homeostasis, cell-cycle control, and survival signaling. Experimental studies demonstrate inhibition of proliferation via cyclin suppression and checkpoint modulation, induction of apoptosis, and attenuation of growth factor–dependent signaling cascades [38,39,67,92]. Importantly, lung tissue is continuously exposed to high oxidant flux; thus, activation of the Nrf2/ARE antioxidant pathway represents a particularly relevant mechanism. Lycopene and its bioactive metabolites, such as apo-10′-lycopenoic acid, have been shown to suppress cyclin E expression, induce cell-cycle arrest, and enhance phase II detoxification enzyme expression via Nrf2 activation, resulting in reduced tumor growth in animal models [38,61,93,94]. These data suggest that lycopene may act not merely as a radical scavenger but as a transcriptional modulator of pulmonary stress-response networks.

Epidemiologically, several case–control studies and pooled analyses report inverse associations between dietary or circulating lycopene and lung cancer risk, with estimated risk reductions frequently in the 20–30% range [1,61,95,96,97,98,99,100,101,102]. Meta-analytic syntheses generally support a protective trend, particularly among populations with higher fruit and vegetable consumption patterns [61,68]. However, findings are not uniformly consistent. Large prospective cohorts, including the VITAL study, have reported no statistically significant association between lycopene intake and lung cancer incidence [103]. Such discrepancies likely reflect heterogeneity in smoking status, baseline oxidative load, exposure misclassification (dietary recall vs. biomarker-based assessment), and the broader dietary matrix in which lycopene is consumed. From a translational perspective, lung cancer illustrates both the strength and limitations of the lycopene evidence base. Mechanistic plausibility is high, particularly in oxidant-driven carcinogenesis, and experimental models show consistent suppression of proliferation and enhancement of antioxidant defense. Yet, human data suggest that any protective effect is likely context-dependent, potentially strongest in individuals with elevated oxidative stress burden or within carotenoid-rich dietary patterns rather than as isolated supplementation [1,61].

#### 3.2.3. Gastric Cancer

Gastric cancer remains a major global health burden, particularly in populations characterized by chronic Helicobacter pylori infection, high-salt diets, nitrosamine exposure, and persistent mucosal inflammation. Dietary patterns rich in fruits and vegetables, including lycopene-containing foods, have repeatedly been associated with reduced gastric cancer risk, suggesting a role for diet-mediated redox and inflammatory modulation in gastric carcinogenesis [104]. Elevated circulating and tissue concentrations of lycopene and related carotenoids have been correlated with lower gastric cancer incidence, plausibly through reinforcement of antioxidant defenses and enhancement of detoxification systems [105]. Experimental studies indicate that lycopene appears to inhibit gastric tumorigenesis through coordinated regulation of oxidative stress, detoxification, and apoptosis. Further studies demonstrate upregulation of glutathione-dependent detoxification enzymes and activation of cytoprotective antioxidant pathways, alongside pro-apoptotic modulation of the Bax/Bcl-2 balance and caspase signaling cascades [106,107]. More specifically, lycopene has been reported to engage both the extrinsic and intrinsic apoptotic pathways, activating the initiator caspases caspase-8 and caspase-9 and the executioner caspase-3, in parallel with an increased Bax/Bcl-2 ratio and reduced expression of the anti-apoptotic proteins Bcl-xL and survivin [67]. Lycopene also exhibits antiproliferative effects in gastric cancer cell lines, suppressing growth factor–related signaling (including EGFR- and COX-2–associated pathways) and attenuating mitogenic transcriptional activity [108]. In vivo models further show reduced tumor growth and improved redox homeostasis following lycopene supplementation, with enhanced chemopreventive effects observed when lycopene is combined with other bioactive compounds, such as S-allylcysteine, supporting a multi-target and potentially synergistic mechanism [39,68,106,107].

Clinical and epidemiological evidence generally aligns with a protective directionality. Several case–control and cohort studies report inverse associations between tomato consumption, serum lycopene levels, and gastric cancer risk, particularly for noncardiac gastric cancer [105,109,110,111,112,113]. However, findings are not entirely consistent, as some studies suggest protective effects primarily for other carotenoids rather than lycopene alone [114], highlighting ongoing heterogeneity in population-based evidence.

#### 3.2.4. Liver Cancer

Liver cancer is a major global malignancy associated with risk factors such as chronic viral hepatitis, liver disease, alcohol use, and aflatoxin exposure [115,116,117]. Phytochemicals, including lycopene, have been investigated for their potential therapeutic and chemopreventive effects in liver cancer. Higher serum lycopene levels have been inversely associated with liver fibrosis severity, and circulating carotenoid levels correlate with hepatic concentrations [118]. Experimental studies demonstrate that lycopene inhibits hepatoma cell metastasis, activates antioxidant pathways such as Nrf2-ARE signaling, reduces oxidative stress and ROS production, and suppresses tumor growth in animal models [43,118,119,120,121]. Lycopene also protects hepatocytes against carcinogen-induced damage, including aflatoxin exposure, and improves antioxidant enzyme activity while reducing inflammatory signaling pathways such as NF-κB and COX-2 and inhibiting Akt/mTOR-related signaling [33,122].

Additional studies show that lycopene reduces preneoplastic lesions, inhibits angiogenesis, migration, and invasion by downregulating MMP-2 and related pathways, and suppresses inflammatory and oncogenic signaling, including NF-κB, STAT3, and IL-6 [123,124,125,126,127,128].

#### 3.2.5. Pancreatic Cancer

Pancreatic cancer is a major cause of cancer-related mortality, with risk factors including chronic pancreatitis, alcohol consumption, smoking, obesity, and antioxidant deficiencies [129,130]. Epidemiological studies have reported inverse associations between lycopene intake or serum levels and pancreatic cancer risk in some populations, including case–control studies showing lower lycopene levels in patients than in controls [131,132,133]. However, findings remain inconsistent, as some studies report protective associations primarily with other antioxidants rather than lycopene itself [129,132].

#### 3.2.6. Colorectal Cancer

Epidemiological evidence suggests an inverse association between lycopene intake, particularly from tomato products, and colorectal cancer risk [134,135]. Experimental studies showed that lycopene inhibits the proliferation of colon cancer cells by inducing cell-cycle arrest, reducing cell viability, and suppressing oncogenic signaling pathways, including Akt and β-catenin [53,136]. Lycopene also inhibits invasion- and metastasis-related processes by modulating MAPK/ERK and PI3K/Akt signaling, reducing MMP expression, and promoting apoptosis through regulation of caspases and Bcl-2 family proteins [137].

Animal studies further support its chemopreventive role, showing reduced aberrant crypt foci formation, decreased tumor growth, and modulation of antioxidant and inflammatory pathways, including Nrf2/HO-1, NF-κB, and COX-2 signaling [138,139]. However, some in vivo studies have reported limited or inconsistent effects depending on treatment timing, sex, or experimental model [140,141]. Clinical and observational studies generally report lower serum lycopene levels in colorectal cancer patients and reduced risk with higher tomato consumption, although some cohort studies have found no significant association, indicating heterogeneity in population-based findings [142,143,144,145,146].

#### 3.2.7. Skin Cancer

Ultraviolet (UV) radiation penetrates different layers of the skin and induces damage through oxidative stress and structural alterations, contributing to skin aging and carcinogenesis [147]. Carotenoids, particularly lycopene, have been shown to mitigate UV-induced skin damage, with studies demonstrating a linear relationship between lycopene intake and reduced erythema formation as well as decreased risk of nonmelanoma skin cancer [23,148,149,150]. Lycopene accumulates in skin tissue, although it is more readily degraded by UV exposure compared with β-carotene, and dietary or supplemental lycopene intake has been associated with reduced sensitivity to UV-induced erythema in humans [151,152,153]. Lycopene also suppresses ROS production, lipid peroxidation, and inflammatory responses while activating Nrf2-mediated antioxidant pathways in skin cancer [154]. However, some studies report no significant effect [155] or opposite effects [156] on skin photocarcinogenesis, suggesting that outcomes may depend on dosage, UV exposure, and experimental conditions.

#### 3.2.8. Head and Neck Cancer

Several studies suggest that lycopene and tomato-based products may have protective and therapeutic potential in head and neck cancers, including laryngeal, oral, and pharyngeal cancers [26,66,157,158]. Animal studies show reduced incidence and progression of squamous cell carcinomas and increased expression of adhesion-related proteins such as E-cadherin and β-catenin following lycopene treatment [125,159,160]. However, human studies are limited; one cohort study reported an inverse association between plasma lycopene, but not dietary lycopene intake, and all-cause mortality in patients with prior head and neck cancer [158].

#### 3.2.9. Prostate Cancer

Prostate cancer is one of the most prevalent malignancies among men worldwide and represents the tumor site for which lycopene has been most extensively investigated [161]. Given the strong contribution of oxidative stress, androgen signaling, and growth factor–mediated proliferation to prostate carcinogenesis, lycopene has emerged as a biologically plausible chemopreventive candidate within this context [1,68]. Lycopene may reduce prostate cancer risk through multiple mechanisms, including scavenging free radicals and preventing DNA damage; modulation of gene expression; inhibition of cancer cell proliferation; induction of apoptosis; and suppression of angiogenesis [162,163,164,165,166,167]. Experimental studies demonstrate inhibition of tumor growth by suppressing Akt-related survival pathways and modulating the PPARγ–LXRα–ABCA1 axis, linking cholesterol homeostasis to prostate cancer progression [39,166,168]. Lycopene has also been shown to attenuate IGF-mediated signaling, reduce proliferation markers such as PCNA, and promote apoptosis by upregulating TP53 and Bax and downregulating Bcl-2, thereby shifting the balance toward growth suppression [61,68,169]. These effects position lycopene as a regulator of both metabolic and oncogenic signaling nodes central to prostate tumor biology.

Epidemiologically, prostate cancer presents one of the most consistent inverse associations with lycopene exposure among solid tumors. Multiple cohort studies and meta-analyses report dose–response relationships between dietary or circulating lycopene levels and reduced prostate cancer risk [1,61,170,171,172]. Several case–control analyses further suggest that higher serum lycopene concentrations are associated with a reduced risk of aggressive or advanced disease [173,174], although not all investigations confirm this association [175], indicating persistent heterogeneity across populations and study designs. Differences in baseline dietary patterns, genetic susceptibility, and biomarker assessment (single plasma measurement versus long-term exposure proxies) likely contribute to variability in effect size.

Clinical intervention studies provide additional, though still intermediate-level, support. Lycopene supplementation has been associated in some patient cohorts with reductions in prostate-specific antigen (PSA), decreased circulating IGF-I concentrations, increased connexin-43 expression, and reductions in tumor burden or proliferation indices [61,68,176,177,178,179]. However, definitive effects on prostate cancer–specific mortality remain unproven, and most trials focus on surrogate biomarkers rather than hard clinical endpoints. A recent randomized controlled trial further suggested that higher lycopene intake may reduce prostate cancer incidence among men with elevated cardiovascular risk, reinforcing the concept that lycopene-rich dietary patterns could exert preventive benefits within metabolically susceptible populations [180].

#### 3.2.10. Renal Cell Carcinoma

Evidence regarding the association between lycopene and renal cell carcinoma (RCC) risk remains inconsistent. Some epidemiological studies report an inverse association between lycopene intake and renal cancer risk, with higher intake linked to reduced incidence, potentially due to its strong antioxidant activity, hydrophobic nature, and ability to modulate transcriptional pathways [31,181,182]. Experimental studies also suggest that lycopene may reduce tumor number and size in animal models, possibly through effects on signaling pathways such as mTOR and EGFR [183].

However, other clinical and observational studies have found no significant association between lycopene intake and RCC risk [183,184]. Additionally, inverse associations between lycopene and renal cancer risk have been reported in non-smokers but not in smokers, indicating potential population-specific effects [185].

#### 3.2.11. Ovarian Cancer

Ovarian cancer and lycopene have been investigated for potential chemopreventive and therapeutic effects. Experimental studies demonstrate that lycopene supplementation reduces tumor incidence, oxidative stress markers, and inflammatory signaling while enhancing antioxidant pathways, including increased Nrf2 and HO-1 expression and reduced NF-κB and STAT3 activity [186]. In animal models, lycopene has also been shown to decrease tumor burden and metastatic load, reduce cancer cell proliferation, lower CA125 expression, and enhance the antitumor efficacy of chemotherapeutic agents such as paclitaxel and carboplatin by modulating EMT- and invasion-related markers [187].

Human evidence remains inconsistent. Some studies report inverse associations between lycopene intake or tomato consumption and ovarian cancer risk, particularly in premenopausal women or in diets rich in fruits and vegetables [188,189], whereas other studies find no significant relationship between dietary or serum lycopene levels and risk [190,191].

**Figure 1:**
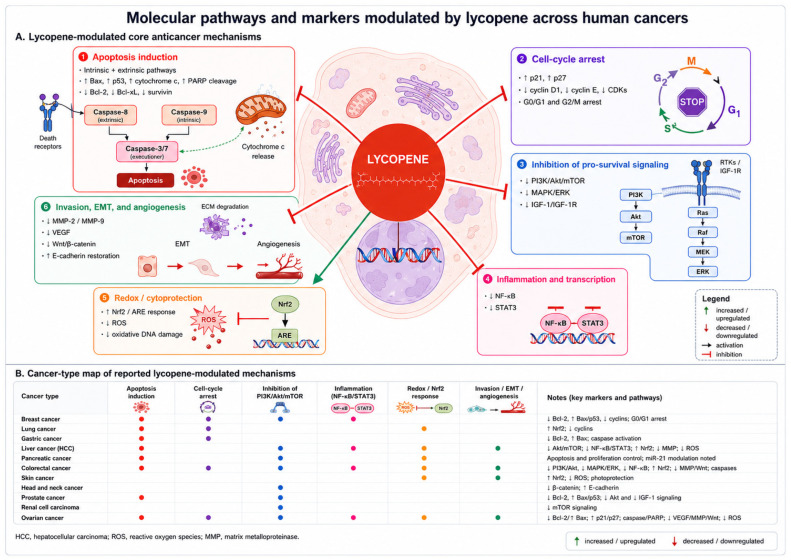
Molecular pathways and markers modulated by lycopene across human cancers. (**A**) Core anticancer mechanisms: apoptosis induction (↑ Bax, ↑ p53, ↑ cytochrome c/PARP; ↓ Bcl-2/Bcl-xL/survivin; caspase-8/-9 → caspase-3/7 cascade), cell-cycle arrest (↑ p21/p27; ↓ cyclins/CDKs), inhibition of pro-survival signaling (PI3K/Akt/mTOR, MAPK/ERK, IGF-1/IGF-1R), reduced inflammation (↓ NF-κB, ↓ STAT3), redox/cytoprotection (↑ Nrf2/ARE; ↓ ROS), and suppression of invasion, EMT and angiogenesis (↓ MMP-2/9, ↓ VEGF, ↓ Wnt/β-catenin; ↑ E-cadherin). (**B**) Reported lycopene-modulated mechanisms across eleven cancers, with key markers per tumor type. ↑, upregulated; ↓, downregulated. HCC, hepatocellular carcinoma; MMP, matrix metalloproteinase; ROS, reactive oxygen species.

Molecular pathways and markers modulated by lycopene across human cancers are summarized in Figure 1.

## 4. Alpha- and Beta-Carotene

Carotenes are polyunsaturated hydrocarbons composed of 40 carbon atoms, containing only carbon and hydrogen, and may include hydrocarbon ring structures at one or both ends of the molecule. β-Carotene is a lipid-soluble orange–yellow carotenoid, which represents a major carotenoid component and serves as a precursor of vitamin A with recognized antioxidant properties [36]. Major sources of β-carotene are green leafy vegetables, carrots, red peppers, broccoli, oranges, and potatoes [192] (Table 1). α-Carotene is structurally similar to β-carotene, differing primarily in the position of double bonds within the terminal hydrocarbon ring [36], and it is a dietary carotenoid with provitamin-A activity that yields one molecule of retinol after central cleavage, with about twice the molar amount needed to match the vitamin A activity of β-carotene [34]. Major sources for α-Carotene are orange carrots and some pumpkins [35] (Table 1).

### 4.1. Molecular Mechanisms of Alpha- and Beta-Carotene in Cancer Prevention

#### 4.1.1. Regulation of Tumor Invasion and Metastasis

Provitamin A carotenoids, particularly α- and β-carotene, exert antitumor activity through multi-target modulation of metastatic signaling networks. Among the carotenes, α-carotene appears to display a comparatively stronger anti-metastatic profile, primarily by suppressing extracellular matrix remodeling and tumor cell motility pathways. Experimental evidence demonstrates that α-carotene inhibits cancer cell invasion, migration, and adhesion by reducing the activity of proteolytic enzymes involved in extracellular matrix degradation, including urokinase plasminogen activator and matrix metalloproteinases MMP-2 and MMP-9. These effects are accompanied by increased expression of metastasis-suppressive proteins, including plasminogen activator inhibitor-1 (PAI-1), tissue inhibitors of metalloproteinases (TIMP-1 and TIMP-2), and NM23-H1. Mechanistically, α-carotene attenuates focal adhesion kinase (FAK) signaling and downstream MAPK pathways (ERK, p38, and JNK), thereby reducing the activation of Rho and Rac GTPases that regulate cytoskeletal organization and tumor cell motility, and limiting metastatic potential [1,193]. Collectively, α-carotene demonstrates the most potent anti-metastatic mechanistic profile among the carotenes, targeting MMP-2/9, focal adhesion kinase, and Rho/Rac GTPase signaling; by contrast, its direct effects on survival pathways such as Akt, NF-κB, and apoptosis are more limited than those of β-carotene or retinoids. By contrast, β-carotene demonstrates anti-invasive activity across a broader range of tumor models, including neuroblastoma, melanoma, colorectal, and gastric cancers. These effects are associated with suppression of MMP-2/9/7/10/28 expression and downregulation of the HIF-1α–VEGF–GLUT1 axis, thereby limiting hypoxia-driven angiogenesis, metabolic adaptation, and metastatic dissemination [1,194,195,196,197,198]. Additionally, β-carotene has been reported to modulate the tumor microenvironment by reducing M2 macrophage polarization and suppressing activated fibroblast signaling, thereby attenuating epithelial–mesenchymal transition (EMT) and cancer stemness features [199].

#### 4.1.2. Regulation of Cell Survival Signaling and Apoptosis

Compared with α-carotene, β-carotene exerts broader effects on tumor cell survival signaling and apoptosis. At physiologically attainable concentrations, β-carotene suppresses pro-survival pathways including NF-κB, Akt, and ERK1/2, thereby reducing proliferation and enhancing programmed cell death [1,13,200,201,202]. These signaling changes are accompanied by decreased expression of anti-apoptotic proteins (Bcl-2, Bcl-xL), increased Bax expression, activation of caspase-3, and PARP cleavage, hallmarks of intrinsic apoptotic pathway engagement [13,200,202]. Notably, dose-dependent redox modulation appears central to β-carotene’s mechanism. While low-to-moderate concentrations often exert antioxidant and cytoprotective effects, higher concentrations in certain tumor contexts may induce controlled pro-oxidant activity, increasing mitochondrial ROS and triggering apoptosis [196,198,202]. β-Carotene also suppresses COX-2/PGE2 signaling, modulates Nrf2/SOD2/HO-1 antioxidant responses, and attenuates stress-adaptive pathways such as XBP-1, thereby weakening tumor survival under metabolic stress [200,201,203]. Importantly, as a provitamin A carotenoid, β-carotene may be cleaved into retinol and subsequently act as a precursor for RAR/RXR-mediated transcriptional regulation, thereby linking carotenoid metabolism to retinoid-dependent differentiation and growth control pathways [1,13].

#### 4.1.3. Modulation of Oncogenic Pathways and Tumor Progression

Comprehensive reviews emphasize that carotenoids, including α- and β-carotene, modulate major oncogenic networks such as PI3K/Akt/mTOR, MAPK, NF-κB, Wnt/β-catenin, and IL-6/STAT3 signaling axes [1,13,198,202]. Within this framework, α-carotene primarily functions as an anti-metastatic regulator of extracellular matrix remodeling and motility signaling, whereas β-carotene exerts broader control over survival signaling, apoptosis, inflammation, and hypoxia-driven metabolic reprogramming [13,202,204]. However, translation to clinical prevention remains complex. While preclinical evidence consistently demonstrates anti-invasive and pro-apoptotic activity, epidemiologic and interventional findings are heterogeneous. High-dose β-carotene supplementation, particularly in smokers, has been associated in some contexts with adverse outcomes, leading to the interpretation that β-carotene may function more reliably as a biomarker of fruit and vegetable intake rather than as a standalone pharmacologic agent [1,13,198]. In contrast, α-carotene has shown comparatively stronger protective signals in certain observational analyses, although mechanistic and clinical data remain more limited [13,205].

### 4.2. Alpha- and Beta-Carotene in Cancer Prevention Across Tumor Types

#### 4.2.1. Breast Cancer

**Figure 2:**
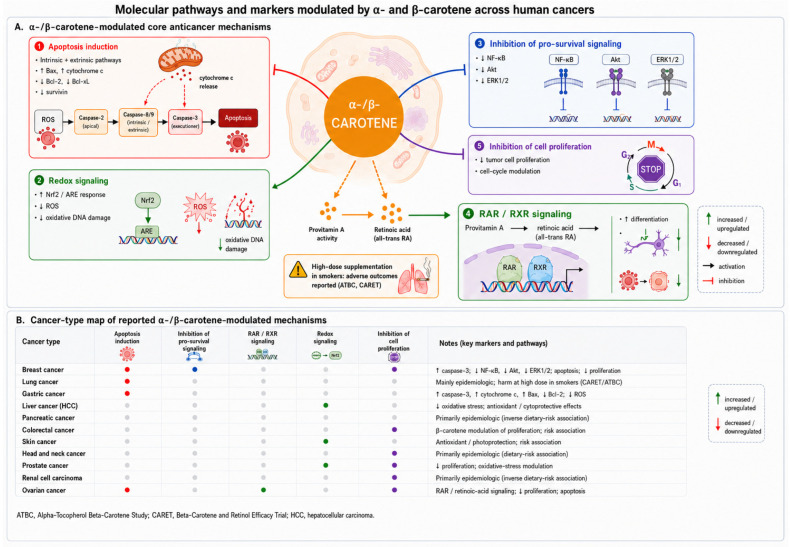
Molecular pathways and markers modulated by α- and β-carotene across human cancers. (**A**) Core anticancer mechanisms: apoptosis induction (↑ Bax/cytochrome c; ↓ Bcl-2/Bcl-xL/survivin; ROS-driven caspase-2/-8/-9 → caspase-3 cascade), redox signaling (↑ Nrf2/ARE; ↓ ROS), inhibition of pro-survival signaling (↓ NF-κB/Akt/ERK1/2), RAR/RXR signaling (provitamin A → retinoic acid → ↑ differentiation, ↓ proliferation), and inhibition of cell proliferation. High-dose supplementation in smokers has been linked to adverse outcomes (ATBC, CARET). (**B**) Reported α-/β-carotene-modulated mechanisms across eleven cancers; several rest mainly on epidemiologic evidence. ↑, upregulated; ↓, downregulated. ATBC, Alpha-Tocopherol Beta-Carotene Study; CARET, Beta-Carotene and Retinol Efficacy Trial; HCC, hepatocellular carcinoma.

The interaction of hormonal milieu, genetic susceptibility, and modifiable lifestyle exposures, including diet and smoking-related oxidative burden, shapes breast cancer risk. Within this framework, provitamin A carotenoids, particularly β-carotene and α-carotene, have been evaluated as candidate protective factors, supported by both mechanistic plausibility and prospective biomarker-based epidemiology [12,78,82,200,206,207,208,209]. At physiologically attainable concentrations, β-carotene has been shown to exert direct antitumor effects in breast cancer cell systems, including induction of apoptosis and suppression of survival signaling. In MCF-7 cells, β-carotene increases apoptotic execution (e.g., caspase-3 activation) while downregulating pro-survival and growth pathways such as NF-κB, Akt, and ERK1/2, accompanied by reductions in anti-apoptotic programs and antioxidant-related markers (Figure 2) [200]. Independent in vitro studies similarly report cell-cycle arrest and apoptosis induction by β-carotene (and, in some settings, lycopene), reinforcing a coherent preclinical signal of growth suppression in breast cancer lines [210]. Human evidence is most consistent when exposure is assessed using circulating carotenoid biomarkers rather than dietary questionnaires. A pooled analysis of 8 prospective studies reported that higher circulating levels of α-carotene and β-carotene were associated with lower breast cancer risk [78]. More recent dose–response syntheses have corroborated inverse associations between circulating carotenoids and breast cancer, suggesting an approximate ~20% risk reduction across higher versus lower biomarker strata in some analyses [209]. Large cohort work also indicates that higher plasma β-carotene and total carotenoids may be more strongly associated with reduced risk of aggressive or lethal breast cancer phenotypes over long follow-up [78], and EPIC biomarker analyses suggest that inverse associations may be more pronounced for ER-negative tumors [211]. Nevertheless, heterogeneity persists. While some meta-analyses of blood β-carotene concentrations report inverse associations [77,206], others do not observe statistically significant relationships [207,212], underscoring between-study differences in populations, endpoints, and analytical control for confounding.

Dietary intake studies generally show weaker associations than biomarker-based studies, likely reflecting measurement error and the difficulty of disentangling single nutrients from overall dietary patterns. However, subgroup signals recur; protective associations may be more evident in smokers or in specific hormonal/metabolic contexts, although interaction tests are not always significant [82,213,214,215]. Importantly, an evidence synthesis incorporating multiple cohorts (and additional designs) reported that higher dietary β-carotene intake was associated with improved breast cancer survival, whereas other provitamin A carotenoids (including α-carotene), β-cryptoxanthin, and retinol did not show consistent survival benefit [208]. An umbrella meta-analysis further supports an overall reduction in breast cancer risk with higher α- and β-carotene exposure, strengthening the inference that circulating carotenoid status may reflect a protective dietary/lifestyle phenotype [12]. Overall, preclinical data support direct antiproliferative and pro-apoptotic actions of β-carotene in breast cancer cells [200,210], while prospective human studies provide the most consistent protective signal for circulating α- and β-carotene [12,78,209]. However, effect sizes are generally modest and partly heterogeneous, and randomized supplementation trials of β-carotene do not reliably reduce cancer risk, reinforcing the view that carotenoids may act as biomarkers or components of carotenoid-rich dietary patterns rather than as isolated high-dose preventive agents [207,216].

#### 4.2.2. Lung Cancer

Several studies have reported an inverse association between α-carotene and lung cancer risk. An animal study found that α-carotene has greater activity than β-carotene in suppressing tumorigenesis in lung cancer [217]. A cohort study demonstrated that higher α-carotene levels were significantly associated with reduced lung cancer risk [218]. Similarly, a prospective cohort study found that higher α-carotene intake was associated with lower risk, with a stronger protective effect observed among never smokers [101]. An additional cohort study also reported a significant reduction in lung cancer risk with higher α-carotene levels [97]. In contrast, a case–control study found no association between α-carotene intake and lung cancer risk [102]. Overall, a meta-analysis further supported the protective effects of α-carotene and retinol against lung cancer [219].

Evidence for β-carotene is more heterogeneous. One cohort study demonstrated a dose-dependent inverse association between dietary β-carotene intake and lung cancer risk [220], and another study found that higher serum β-carotene levels were associated with reduced risk [221]. A separate cohort study similarly reported a significant inverse association [97]. Critically, the CARET (Beta-Carotene and Retinol Efficacy Trial) was stopped early because β-carotene supplementation combined with retinyl palmitate led to a 28% increase in lung cancer incidence and a 17% increase in overall mortality among smokers and asbestos-exposed workers compared to placebo, the opposite of the intended chemopreventive effect [222]. The ATBC (Alpha-Tocopherol, Beta-Carotene Cancer Prevention) trial similarly demonstrated an 18% higher cumulative lung cancer incidence among male smokers who received β-carotene relative to those who did not, accompanied by an 8% excess in overall mortality [223]. These findings represent a landmark cautionary result: the protective associations observed with dietary β-carotene intake do not translate to, and may be counteracted by, isolated high-dose supplementation in individuals with significant oxidative stress from smoking or asbestos exposure. In addition, a cohort study observed an inverse but non-significant relationship [218], and a case–control study found no association [102]. Notably, four meta-analyses reported that β-carotene intake was associated with an increased risk of lung cancer [207,224,225,226], whereas an umbrella analysis did not demonstrate a statistically significant association between β-carotene and lung cancer risk [12].

#### 4.2.3. Gastric Cancer

There are limited studies evaluating the relationship between carotenoids and gastric cancer. Low-dose β-carotene supplementation has been associated with reduced gastric cancer incidence in Chinese populations, and another study reported lower stomach cancer rates among individuals receiving combined supplementation with β-carotene, vitamin E, and selenium [227]. In contrast, higher-dose β-carotene supplementation was associated with an increased risk of gastric cancer in smokers and asbestos-exposed individuals [207]. Additionally, another study found no significant effect of β-carotene supplementation on gastric cancer progression [228].

#### 4.2.4. Liver Cancer

From an HCC perspective, the most reproducible epidemiologic signal points to retinol (vitamin A) insufficiency as a strong risk marker, potentially reflecting both impaired hepatic storage/transport and a biologically relevant vulnerability to chronic inflammation and oxidative injury in the cirrhotic liver. By contrast, α- and β-carotene appear more consistently as susceptibility modifiers that interact with smoking, alcohol exposure, and host detoxification capacity, rather than as independent, direct protective agents [193,229,230,231,232,233,234,235,236,237,238,239]. Case–control evidence has reported that low retinol concentrations are associated with markedly higher odds of HCC, on the order of ~fivefold in some analyses, whereas β-carotene was not statistically significant in the same models [229]. Parallel clinical observations in high-risk liver disease populations support this pattern: patients with chronic liver disease who develop HCC tend to have lower baseline serum retinol than comparators [231,238], consistent with the concept that retinoid deficiency may promote hepatocarcinogenesis or reflect a permissive milieu for malignant transformation in cirrhosis [231]. Prospective data from Taiwan and Shanghai likewise reported reduced liver cancer risk among individuals with higher prediagnostic serum retinol levels [233,234]. Importantly, Mendelian randomization analyses further suggest that genetically proxied perturbations in retinol metabolism may causally increase HCC risk, strengthening the biological plausibility beyond confounding and reverse causation [240]. Several observational analyses do not support a robust, independent association between plasma α-/β-carotene and HCC risk when examined in multivariable models [230]. However, mechanistically coherent interaction signals have been reported: low carotenoid status may amplify smoking- and alcohol-related HCC risk, particularly in individuals with reduced detoxification capacity such as the GSTM1 null genotype, implying that carotenoids may buffer carcinogenic/oxidative exposures rather than directly preventing tumor initiation [230]. This framing is also consistent with studies in high-risk HCV cohorts in which carotenoid status declines with disease severity and inversely tracks oxidative stress burden, suggesting that low circulating carotenoids may represent both increased utilization and impaired hepatic handling [118].

Randomized evidence does not convincingly support β-carotene–based supplementation for preventing liver cancer outcomes. In an intervention setting, β-carotene combined with α-tocopherol and selenium did not significantly reduce liver cancer mortality overall [232]. More broadly, large-scale antioxidant supplementation meta-analyses have not demonstrated consistent protection against gastrointestinal cancers and have raised concerns about neutrality or harm depending on population risk profiles and dose context [241,242]. In the ATBC cohort, observational baseline status of retinol/β-carotene has been linked to subsequent liver cancer incidence and chronic liver disease mortality, yet supplementation itself did not translate into a robust preventive signal, again consistent with a “status marker vs. pill effect” separation [237,239]. At the cellular level, direct comparisons suggest that α-carotene can suppress invasion, migration, and adhesion of human hepatocarcinoma cells more effectively than β-carotene at similar concentrations [193]. This aligns with the broader mechanistic literature that positions α-carotene as relatively stronger in anti-invasive/anti-metastatic phenotypes, whereas β-carotene may act more variably across survival/redox pathways depending on dose and cellular context. The HCC literature supports a model in which low retinol represents a high-value risk indicator, potentially mechanistically relevant and/or reflective of advanced hepatic dysfunction, while α-/β-carotene shows weaker independent associations but may meaningfully modify susceptibility under high oxidative/carcinogenic exposures [229,230,231,232,233,234,235,236,237]. Translationally, these data argue for prioritizing (i) careful assessment and correction of clinically significant vitamin A deficiency in chronic liver disease where appropriate, and (ii) focusing on carotenoids within whole-diet patterns and exposure-stratified prevention models, rather than relying on single-agent β-carotene supplementation to reduce HCC mortality [232,239,241].

#### 4.2.5. Pancreatic Cancer

An epidemiologic study reported an inverse association between vitamin A and β-carotene levels and the risk of pancreatic cancer [132], and a meta-analysis supported a protective effect of higher β-carotene intake [243]. Similarly, a prospective study suggested that greater intake of retinol activity equivalents and β-carotene equivalents was associated with reduced pancreatic cancer risk among overweight individuals [244]. In contrast, a case–control study did not observe any association between intake of α-carotene and β-carotene and pancreatic cancer risk [245]. A systematic meta-analysis likewise reported no significant effect of β-carotene supplementation on pancreatic cancer incidence [207], a finding echoed by additional meta-analyses [207,225]. Consistently, a randomized controlled trial also showed no significant impact of β-carotene supplementation on pancreatic cancer incidence [246].

#### 4.2.6. Colorectal Cancer

The evidence base for β-carotene and colorectal cancer (CRC) is dominated by randomized trials and large prospective cohorts that largely indicate a neutral association, whereas more recent case–control studies and analyses of broader carotenoid exposure (including α-carotene and total carotenoids) occasionally suggest modest protective signals. A population-based analysis reported no association between β-carotene exposure and colorectal cancer risk [247]. Consistent with this, a controlled trial in older male smokers found no evidence of benefit or harm from β-carotene supplementation on CRC incidence [248]. The Finnish ATBC trial (29,133 male smokers; 20 mg/day β-carotene) similarly showed no meaningful effect on colorectal cancer outcomes (RR ≈ 1.05), supporting an overall “neutral” trial-level signal [248]. Meta-analytic syntheses of randomized supplementation trials further reinforce this conclusion, reporting no significant reduction in colorectal/colon cancer risk with β-carotene (and no consistent benefit for vitamin A/retinol supplementation for colon cancer prevention) [207,249,250]. Importantly, broader trial meta-analyses emphasize that high-dose β-carotene supplementation does not confer generalized cancer protection and may increase risk for certain cancers in specific high-risk groups (e.g., smokers), underscoring the context-dependence of provitamin A carotenoid supplementation [207,250].

Large prospective investigations have generally not supported a robust protective association between dietary β-carotene intake and CRC. A pooled analysis of 11 cohort studies found no significant relationship between dietary carotenoids (including α- and β-carotene) and colorectal cancer risk [251]. Similarly, the Multiethnic Cohort reported no clear association between carotenoid intake and colorectal cancer incidence [252], and EPIC analyses evaluating both plasma and dietary carotenoids likewise observed largely null or weak associations across colon and rectal endpoints [253]. Meta-analyses of observational studies have generally aligned with these findings, reporting no statistically significant association between β-carotene intake and colon cancer risk [207,225,254], although some syntheses note a small, non-significant trend toward risk reduction [254]. An umbrella meta-analysis suggests that “serum total carotenoids” may show a clearer inverse association than individual dietary carotenoids, again pointing to biomarker-based exposure assessment as potentially more informative than dietary recall [12].

In contrast to the predominantly neutral trial and cohort evidence for β-carotene, several case–control studies, particularly in Mediterranean and Asian settings, have reported stronger inverse associations for α-carotene, β-carotene, or total carotenoid intake with CRC risk [255,256,257]. A recent case–control study specifically reported a protective association between colon cancer and higher intake of α-carotene and β-carotene [258]. More recently, an Italian case–control analysis found inverse associations between total and selected carotenoids and colorectal cancer risk, supporting the hypothesis that carotenoid-rich dietary patterns may contribute to protection in certain populations [258]. Complementing incidence data, a cross-sectional study and meta-analysis reported that higher dietary β-carotene/vitamin A intake was associated with lower prevalence of colorectal adenoma, suggesting a possible role earlier in the adenoma–carcinoma sequence [259]. Taken together, the CRC literature indicates that high-dose β-carotene supplementation is largely ineffective (“neutral”) for CRC prevention in randomized evidence [207,248,250]. Observational data remain heterogeneous: large cohorts often show weak or null associations [251,252,253], whereas selected case–control studies report more pronounced protective associations, particularly for α-carotene and total carotenoids [255,258]. This divergence is consistent with methodological and biological explanations, including exposure misclassification in dietary questionnaires, differences in baseline dietary patterns, smoking status, and redox milieu, and the likelihood that carotenoids act as components or biomarkers of broader plant-rich dietary patterns rather than as single-agent supplements [12,254]. Consequently, current evidence supports prioritizing carotenoid-rich whole-food approaches over isolated β-carotene supplementation for CRC chemoprevention research.

#### 4.2.7. Skin Cancer

β-Carotene has been widely reported to exert photoprotective effects [260,261,262,263], and animal studies suggest that carotenoids may protect against UV-induced skin cancer [264]. A cross-sectional study found that higher serum β-carotene levels were associated with less severe sunburn, whereas higher vitamin A levels correlated with greater sunburn severity, which in turn was linked to increased cancer risk [227]. A cohort study also reported that higher dietary vitamin A intake was associated with reduced risk of cutaneous squamous cell carcinoma [265].

However, other evidence is less supportive. Retrospective and prospective studies found no association between skin or plasma carotenoid levels and nonmelanoma skin cancer [266,267]. Randomized trials similarly showed no protective effect of β-carotene supplementation on nonmelanoma skin cancer [267,268,269]. A systematic meta-analysis confirmed that β-carotene supplementation had no significant effect on melanoma or nonmelanoma skin cancer risk [207], and long-term supplementation did not reduce the incidence of basal cell carcinoma or squamous cell carcinoma, nor were baseline plasma carotenoid levels associated with risk [270].

#### 4.2.8. Head and Neck Cancer

In case–control studies, inverse associations were observed between α- and β-carotene intake and laryngeal [271,272] and oral cavity cancers [273], and a pooled analysis of 10 case–control studies reported inverse relationships between total carotenoid intake and oral/pharyngeal and laryngeal cancers, with β-carotene equivalents showing the strongest effect [274]. However, one case–control study found no significant associations with α- or β-carotene intake [275], and no association was observed with total vitamin A or retinol intake [271]. A large prospective cohort study did not demonstrate significant associations between α-carotene, β-carotene, and head and neck cancer risk [276]. Among non-smokers, higher α-carotene and total carotenoid levels were associated with reduced mortality, whereas elevated plasma retinol was linked to increased mortality in smokers [158]. A meta-analysis further showed that higher consumption of β-carotene equivalents and α-carotene was associated with a lower risk of oral cavity and pharyngeal cancers [277].

In a randomized interventional study, β-carotene or vitamin A supplementation did not significantly reduce oral lesion progression or oral squamous cell carcinoma [278], and a meta-analysis [279] confirmed this. Additionally, randomized trials demonstrated no significant benefit of β-carotene supplementation in reducing second primary tumors or recurrence, nor in improving disease-free or overall survival among patients with early-stage head and neck squamous cell carcinoma [280,281]. Although high-dose isotretinoin followed by low-dose maintenance improved lesion stability compared with β-carotene [282], and vitamin A, alone or combined with β-carotene, resulted in higher remission rates and prevention of new oral leukoplakia [283,284], a large chemoprevention trial concluded that 13-cis retinoic acid and retinyl palmitate, alone or with β-carotene, cannot be recommended for chemoprevention [285].

#### 4.2.9. Prostate Cancer

Preclinical studies demonstrated that high-dose β-carotene reduced the proliferation of prostate cancer cells in vitro [286,287]. In case–control analyses, higher circulating levels of α-carotene and trans-β-carotene were associated with lower prostate cancer risk, although not with disease progression [288]. A retrospective study also reported that β-carotene supplementation in men with low dietary intake was associated with reduced prostate cancer risk [289]. In contrast, a nested case–control study found that β-carotene was associated with an increased risk of aggressive prostate cancer [290], and another prospective case–control study showed no protective effect of β-carotene or retinol [291]. A retrospective analysis further indicated that serum β-carotene, serum retinol, and supplemental β-carotene had no apparent effect on prostate cancer survival [288].

Prospective studies yielded inconsistent findings: one study reported no associations between plasma carotenoids, retinol, or tocopherols and overall prostate cancer risk [292], whereas another observed a protective effect of dietary β-carotene and vitamin A [293]. Meta-analyses of randomized controlled trials showed no overall effect of β-carotene supplementation on prostate cancer risk [207,225]. Individual randomized trials were also conflicting: supplementation reduced prostate cancer incidence among men with the highest BMI [294], while another controlled trial reported higher prostate cancer incidence and mortality among those receiving β-carotene [295].

Notably, the ATBC prostate subanalysis demonstrated that α-tocopherol supplementation reduced prostate cancer incidence by 32% among male smokers, whereas β-carotene supplementation showed no protective effect and was associated with numerically higher prostate cancer mortality, reinforcing the complexity of carotenoid effects in high-oxidative-stress populations [295].

#### 4.2.10. Renal Cell Carcinoma

In case–control studies, significant inverse associations were observed between α-carotene and β-carotene intake and renal cell carcinoma (RCC) risk [296], and a diet rich in β-carotene was suggested to contribute to RCC prevention [297].

Prospective findings have been inconsistent. A pooled analysis of prospective studies reported that β-carotene intake was associated with a decreased risk of RCC, whereas α-carotene showed no significant effect [298]. One prospective study found a reduced RCC risk in men with higher vitamin A intake [184], whereas another prospective cohort study did not observe a protective association with dietary β-carotene or α-carotene intake [181]. In a randomized trial, neither α-tocopherol nor β-carotene supplementation affected the incidence of urothelial or renal cancer [299].

#### 4.2.11. Ovarian Cancer

Case–control and retrospective studies have reported predominantly inverse associations between carotenoids and ovarian cancer risk. A retrospective study found that higher intake of carotene, particularly α-carotene, from food and supplements was significantly associated with reduced ovarian cancer risk, mainly among postmenopausal women [188]. Case–control studies also suggested modest protective effects of vitamin A and β-carotene, especially among smokers [300], and reported protective roles for both α- and β-carotene [301]. However, another case–control study found no association between β-carotene intake and ovarian cancer risk [302].

Prospective and pooled cohort analyses have yielded inconsistent findings. A pooled cohort analysis suggested that adult consumption of β-carotene or α-carotene does not play a major role in ovarian cancer incidence [303], and a prospective study found no association between vitamin A intake and reduced ovarian cancer risk [304]. In contrast, a meta-analysis indicated that high dietary β-carotene intake may confer a modest protective effect [305]. Additionally, a Mendelian randomization analysis reported that genetically higher β-carotene concentrations were associated with an increased risk of invasive epithelial ovarian cancer [306].

Molecular pathways and markers modulated by carotenoids across human cancers are summarized in Figure 2.

## 5. Retinoids

Retinoids comprise a structurally diverse class of natural and synthetic vitamin A-related compounds with uniquely broad transcriptional activity in cancer biology. Endogenous retinoids include retinol, retinal, all-trans retinoic acid (ATRA), and 9-cis retinoic acid, whereas clinically used synthetic analogs include isotretinoin (13-cis retinoic acid), acitretin, bexarotene, and fenretinide (4-HPR) [307,308,309,310,311]. Their primary biological effects are mediated through two nuclear receptor families, retinoic acid receptors (RARα/β/γ) and retinoid X receptors (RXRα/β/γ), which form homo- or heterodimers and bind retinoic acid response elements (RAREs) in target gene promoters, thereby regulating transcriptional programs controlling proliferation, differentiation, apoptosis, and invasion [307,308,309,311,312,313]. In this respect, retinoids activate a more direct and expansive nuclear transcriptional network than non–provitamin A carotenoids such as lycopene, whose anticancer effects are typically mediated indirectly via redox and signaling modulation rather than canonical RAR/RXR-driven differentiation programs. Retinoids thus engage the broadest nuclear transcriptional program among the compounds reviewed, activating RAR/RXR heterodimers that regulate differentiation, apoptosis (via cyclin D1/E suppression and Bcl-2 family modulation), and anti-invasive signaling (via MMP-2/9 and NF-κB inhibition), pathways largely absent or indirect in non-provitamin A carotenoids such as lycopene [307,308,309,311].

### 5.1. Mechanisms of Retinoid Anticancer Activity

**Figure 3:**
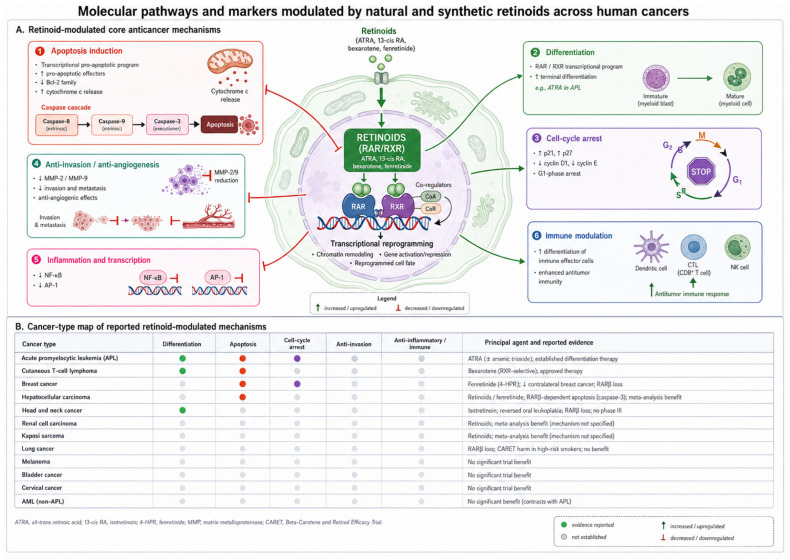
Molecular pathways and markers modulated by natural and synthetic retinoids across human cancers. (**A**) Core mechanisms driven by ligand-activated RAR/RXR transcriptional reprogramming: apoptosis induction (↓ Bcl-2 family; cytochrome c release; caspase-8/-9 → caspase-3 cascade), differentiation (e.g., ATRA in APL), cell-cycle arrest (↑ p21/p27; ↓ cyclin D1/E; G1 arrest), anti-invasion/anti-angiogenesis (↓ MMP-2/9), reduced inflammation (↓ NF-κB/AP-1), and immune modulation. (**B**) Reported retinoid-modulated mechanisms across the tumor types in Section 5, with principal agent and clinical evidence; filled circles denote reported evidence, gray denotes not established. ↑, upregulated; ↓, downregulated. ATRA, all-trans retinoic acid; 4-HPR, fenretinide; APL, acute promyelocytic leukemia; MMP, matrix metalloproteinase.

Mechanistically, retinoids promote cell differentiation and suppress proliferation by inducing G1-phase arrest, classically through upregulation of cyclin-dependent kinase inhibitors (p21, p27) and downregulation of cyclin D1 and cyclin E [308,311,312,314]. They also promote apoptosis by transcriptional activation of pro-apoptotic effectors and repression of anti-apoptotic programs, including modulation of the Bcl-2 family balance and caspase-linked pathways [308]. Consistent with this, specific retinoids engage both apoptotic arms: the synthetic retinoid fenretinide (4-HPR) can activate the initiator caspase-8 in a death receptor-independent manner and trigger cytochrome c release with caspase-9 activation, converging on the executioner caspase-3, while caspase-2 acts as an apical caspase in the broader apoptotic program [315,316] (Figure 3). RARβ loss, commonly observed in head and neck, lung, and breast cancers, is associated with retinoid resistance and has been proposed as a biomarker of chemopreventive response [317]. Retinoids also interfere with NF-κB and AP-1 transcription factor pathways, contributing to anti-inflammatory and anti-invasive effects, and can modulate immune function by promoting the differentiation of immune effector cells [314].

### 5.2. Clinical Use of Retinoids in Cancer

Clinically, ATRA is the prototypical success story of retinoid oncology: in acute promyelocytic leukemia (APL), ATRA-based differentiation therapy (often combined with arsenic trioxide or anthracycline-based regimens) induces high complete remission rates and forms a cornerstone of modern APL management [310,318,319]. This clinical success, however, has not yet translated broadly into solid-tumor chemoprevention.

In head and neck chemoprevention, isotretinoin demonstrated activity in reversing oral leukoplakia and reducing second primary tumors in early-stage disease [280,282,285], but sustained benefit has not been demonstrated in definitive phase III trials, and the toxicity profile (teratogenicity, mucocutaneous effects, hyperlipidemia) limits long-term use [320]. The large Euroscan trial and related studies have not established retinoids as standard-of-care chemopreventive agents in head and neck or lung cancer [321]. In lung cancer, trials combining β-carotene with retinyl palmitate (CARET) demonstrated harm rather than benefit in high-risk populations. In breast cancer, fenretinide (4-HPR), a synthetic retinoid, demonstrated a significant reduction in contralateral breast cancer in premenopausal women in Italian randomized trials, though overall survival benefit was not established [322,323]. Bexarotene, an RXR-selective retinoid (rexinoid), has received approval for the treatment of cutaneous T-cell lymphoma [324]. In summary, retinoids represent a clinically validated class of anticancer agents in specific settings, most notably APL and cutaneous T-cell lymphoma, but their use in solid-tumor chemoprevention is limited by toxicity and inconsistent efficacy [320,321]. The identification of RARβ methylation and other biomarkers of retinoid sensitivity may enable future precision-based approaches to patient selection [317]. A broader discussion of epigenetic mechanisms, including DNA methylation, histone modifications, and miRNA regulation, is provided in Section 6.

A recent meta-analysis of randomized trials showed that retinoids resulted in improved overall survival, cancer development, disease progression, or event-free survival in APL, renal cell carcinoma, hepatocellular carcinoma, Kaposi sarcoma, and some lung cancers, but had no significant benefit in head and neck cancer, AML, melanoma, breast, bladder, or cervical cancers. Overall, benefits were more pronounced in solid tumors than in hematologic malignancies [320].

Molecular pathways and markers modulated by retinoids across human cancers are summarized in Figure 3.

## 6. Epigenetic Mechanisms of Carotenoids and Retinoids in Cancer Chemoprevention

Beyond their effects on redox balance, signal transduction, and nuclear receptor activity, carotenoids and retinoids also appear to act on the cancer epigenome, influencing DNA methylation, histone modifications, and microRNA (miRNA) expression. Because these marks are reversible, unlike genetic mutations, they represent an appealing target for dietary chemoprevention and may help explain how sustained dietary exposure translates into lasting changes in gene expression.

### 6.1. DNA Methylation

Lycopene has been shown to reverse aberrant promoter hypermethylation of the tumor suppressor gene GSTP1 in androgen-independent PC-3 prostate cancer cells, restoring GSTP1 mRNA and protein expression while lowering DNMT3A levels, although the same effect was not seen in androgen-dependent LNCaP cells [325]. In a cohort of head and neck cancer survivors, circulating lycopene levels were linked to distinct leukocyte DNA methylation patterns within inflammatory signaling pathways, suggesting that lycopene status leaves a measurable epigenetic signature relevant to long-term outcomes [326]. Beta-carotene produced comparable effects in colon cancer stem cells, reducing DNMT3A expression and global DNA methylation alongside its antiproliferative activity [327].

Among the retinoids, silencing of the tumor suppressor gene RARβ2 by CpG island hypermethylation is one of the most consistently reported epigenetic events in solid tumors. Meta-analyses indicate that RARβ2 promoter methylation is roughly seven times more common in breast cancer tissue than in non-cancerous controls [328], and a similar association has been reported for prostate cancer [329], supporting RARβ2 methylation as a candidate biomarker of retinoid sensitivity, a point raised elsewhere in this review.

### 6.2. Histone Modifications

Histone deacetylation frequently accompanies, and can independently drive, RARβ2 silencing. In cervical cancer cells, RARβ2 repression was traced to either DNA methylation or histone deacetylation, depending on the cell line, and a histone deacetylase (HDAC) inhibitor was able to restore RARβ2 expression even when the promoter itself remained unmethylated, pointing to histone acetylation status as an independent determinant of retinoid responsiveness [330]. Beta-carotene likewise increased histone H3 and H4 acetylation in colon cancer stem cells, an effect that occurred together with reduced DNA methylation and altered miRNA expression, suggesting that this carotenoid acts on multiple layers of the epigenome in parallel rather than through a single mechanism [327].

### 6.3. MicroRNAs

Carotenoids and retinoids also shape gene expression through miRNAs. Lycopene reduced viability, migration, and reactive oxygen species levels in pancreatic cancer cells, with stronger effects in cells lacking the oncogenic miR-21, indicating that miRNA status partly determines lycopene sensitivity [331]. In colon cancer stem cells, beta-carotene altered a panel of miRNAs linked to its histone acetylation effects, again tying miRNA regulation to broader chromatin changes [327].

Retinoids show some of the most extensively documented miRNA effects among the compounds covered in this review. A systematic review of all-trans retinoic acid (ATRA)-induced miRNA changes across neoplastic cell lines found consistent, dose- and duration-dependent shifts in oncogenesis-related miRNAs [332]. In acute promyelocytic leukemia, ATRA-driven differentiation is accompanied by upregulation of miR-15a, miR-15b, miR-16-1, several let-7 family members, miR-223, miR-342, and miR-107, along with downregulation of miR-181b; miR-107 was shown to directly target NFI-A, a transcription factor involved in granulocytic differentiation [333].

## 7. The Supplementation Paradox: Dietary Carotenoids Versus Isolated Supplementation

One of the most important lessons from carotenoid research is the divergence between the protective associations observed with dietary carotenoid-rich food patterns and the frequently null or harmful effects seen with isolated high-dose supplementation. This supplementation paradox has been most dramatically demonstrated with β-carotene in lung cancer but reflects a broader principle that merits explicit discussion.

Several factors likely account for this divergence. First, carotenoids in whole foods act within a complex matrix of synergistic phytochemicals, fiber, vitamins, and minerals, and the observed inverse associations in dietary studies may reflect overall diet quality rather than carotenoid-specific effects [334]. Second, the isomer composition of carotenoids differs fundamentally between food sources (predominantly all-trans) and supplements (variable isomer profiles), affecting bioavailability and receptor interactions. Third, high-dose supplementation may shift carotenoid metabolism toward pro-oxidant pathways, particularly in the presence of high oxidative stress from smoking or carcinogen exposure; in this context, excess β-carotene may generate cleavage products that act as antagonists of RAR signaling and promote rather than suppress carcinogenesis [335,336]. Fourth, epidemiological studies of dietary carotenoid intake are systematically confounded by the fact that high-fruit-and-vegetable consumers differ from low consumers across dozens of health behaviors that are incompletely controlled even in large prospective cohorts [337].

This context is essential for interpreting the clinical evidence reviewed throughout this manuscript. The inverse dietary associations observed for lycopene and prostate cancer, or for carotenoids and breast cancer, should be understood as associations with a dietary pattern rather than as evidence supporting supplementation with specific carotenoids. Any clinical application of carotenoids as chemopreventive agents must be tested prospectively in well-designed randomized trials with appropriate dose, isomer, and population selection, rather than extrapolated from dietary epidemiology.

**Figure 4:**
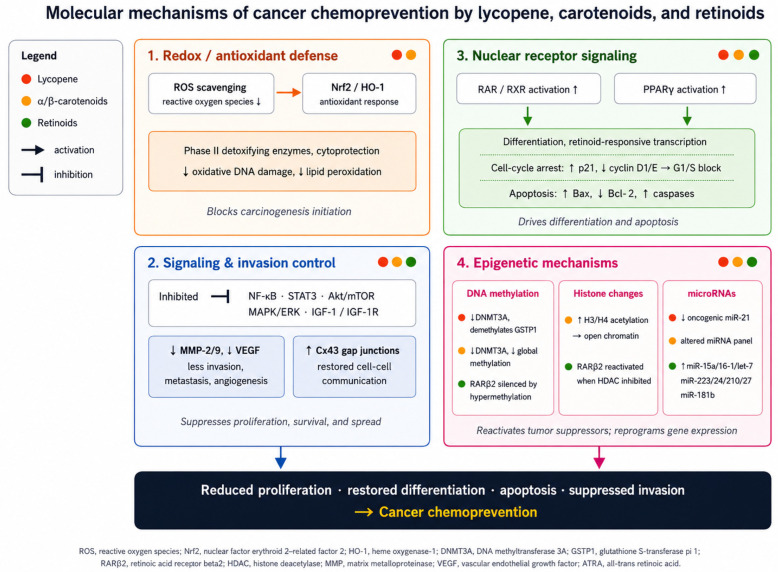
Molecular mechanisms of cancer chemoprevention by lycopene, carotenoids, and retinoids. Four converging categories, color-coded by compound (red, lycopene; amber, α-/β-carotenoids; green, retinoids): (**1**) redox/antioxidant defense (ROS scavenging, Nrf2/HO-1, phase II enzymes); (**2**) signaling and invasion control (↓ NF-κB/STAT3/Akt/mTOR/MAPK-ERK/IGF-1; ↓ MMP-2/9, ↓ VEGF; ↑ Cx43); (**3**) nuclear receptor signaling (RAR/RXR and PPARγ → differentiation, cell-cycle arrest, apoptosis); and (**4**) epigenetic mechanisms (DNA methylation, histone modifications, microRNAs), together reducing proliferation, restoring differentiation, promoting apoptosis and suppressing invasion. ↑, upregulated; ↓, downregulated. HO-1, heme oxygenase-1; MMP, matrix metalloproteinase; Nrf2, nuclear factor erythroid 2–related factor 2; ROS, reactive oxygen species; VEGF, vascular endothelial growth factor.

Molecular mechanisms of cancer chemoprevention by lycopene, carotenoids, and retinoids are summarized in Figure 4.

## 8. Future Perspectives and Conclusions

Future research on carotenoids and retinoids in cancer prevention should move beyond isolated high-dose supplementation trials and instead focus on precision-based, mechanism-driven strategies. Integrating molecular biomarkers, including RARβ methylation status for retinoids, Nrf2 pathway activity for lycopene, and carotenoid cleavage enzyme polymorphisms (BCO1, BCO2), alongside redox profiling and genetic susceptibility factors, may help identify subpopulations most likely to benefit. Greater emphasis should be placed on whole-food dietary patterns rather than single-nutrient interventions, recognizing the synergistic interactions among phytochemicals within complex food matrices. Standardization of dosing, isomer composition (particularly the ratio of all-trans to cis-lycopene), and bioavailability assessment, along with longer follow-up and tumor subtype–specific analyses, will be critical for resolving current inconsistencies. The development of tissue-based carotenoid biomarkers, given the demonstrated discrepancy between plasma and tissue levels, is an important methodological priority. In addition, well-designed translational studies linking epidemiologic observations with mechanistic endpoints, such as oxidative stress markers, inflammatory mediators, and pathway-specific signaling changes, are needed to clarify causality. For retinoids, the identification of predictive biomarkers of response warrants integration into prospective chemopreventive trials, and combination strategies pairing retinoids with carotenoids warrant investigation as a potentially more promising approach than monotherapy, given their complementary mechanisms of action, although direct clinical evidence for this strategy remains limited. The mechanistic diversity across carotenoids and retinoids, spanning antioxidant, nuclear receptor, kinase, and gap junction pathways, is systematically compared in Table 2, which underscores the complementary rather than redundant pathway coverage of these compound classes.

In conclusion, carotenoids and retinoids possess biologically plausible anticancer properties, supported by extensive experimental evidence; however, clinical outcomes remain heterogeneous and context dependent. While dietary intake of carotenoid-rich foods is generally associated with reduced cancer risk across multiple tumor types, high-dose supplementation has not consistently demonstrated benefit and has caused harm in certain high-risk populations, most notably the increased lung cancer risk from β-carotene supplementation in smokers and asbestos-exposed individuals observed in the CARET and ATBC trials. A shift toward personalized nutrition approaches, biomarker-guided interventions, whole-food dietary strategies, and appropriately powered clinical trials is essential to define the true chemopreventive potential of carotenoids and retinoids in human cancer. Patients and clinicians should be specifically cautioned against the assumption that supplements are equivalent to dietary sources, and against high-dose β-carotene supplementation in current or former smokers, as detailed in the clinical trial evidence summarized in Table 3 appended to this manuscript.

## Figures and Tables

**Table 1:** Principal natural and dietary sources of lycopene, α- and β-carotene, and retinoids.

Compound	Main Class	Key Natural/Dietary Sources
Lycopene	Non–provitamin A carotenoid	Tomatoes and processed tomato products (paste, sauce, juice, ketchup), watermelon, pink guava, pink grapefruit, papaya; very high levels in gac (Momordica cochinchinensis) [23,24,25]
α-Carotene	Provitamin A carotenoid	Carrots, pumpkin/winter squash, sweet potato, orange-fleshed vegetables; smaller amounts in green leafy vegetables (spinach, kale) [34,35]
β-Carotene	Provitamin A carotenoid	Carrots, sweet potato, pumpkin/winter squash, dark leafy greens (spinach, kale, collard greens), red/orange bell peppers, apricots, cantaloupe [36]
Retinoids (preformed vitamin A)	Vitamin A derivatives (retinol, retinyl esters, retinoic acid)	Not present in plants. Obtained from animal-derived foods: liver and liver products, fish and fish-liver oils, egg yolk, and dairy (milk, butter, cheese); also fortified foods [37]

**Table 2:** Mechanistic Pathways Modulated by Carotenoids and Retinoids in Cancer.

Pathway/Target	Lycopene	α-Carotene	β-Carotene	Retinoids	Cancer Relevance	Key Refs
Antioxidant/ROS scavenging	+++	++	++	+	Oxidative DNA damage, lipid peroxidation, carcinogenesis initiation	[36,46,47,48,338]
Nrf2/HO-1 activation	+++	++	++	+	Cytoprotection, phase II enzyme induction, detoxification	[40,41,42,43,44]
NF-κB inhibition	+++	++	+	+++	Inflammation, tumor promotion, invasion, metastasis	[59,60,166,308]
STAT3 inhibition	++	+	+	++	Oncogenic transcription, cytokine-driven proliferation	[123,128,186]
Akt/mTOR suppression	++	+	+	++	Cell survival, proliferation, angiogenesis, drug resistance	[53,54,308]
Wnt/β-catenin modulation	+	+	+	+++	Stem-cell renewal, colorectal carcinogenesis, EMT	[53,136,308]
MAPK/ERK signaling	++	+	+	+++	Differentiation, proliferation, invasion	[166,193,308]
Apoptosis (Bcl-2/Bax, caspases)	+++	++	++	+++	Tumor cell death, chemosensitisation	[50,51,52,66,69,70,72,73,308]
Cell-cycle arrest (cyclin D1/E, p21)	++	+	+	+++	G1/S phase block, antiproliferative	[50,51,52,166,308]
RAR/RXR nuclear signaling	−	+	++	+++	Differentiation, retinoid-responsive gene transcription	[166,307,308,309]
PPARγ activation	++	+	+	++	Anti-inflammatory, adipogenic, antitumor signaling	[166]
Gap junction/Cx43 upregulation	+++	++	+	+	Intercellular communication, tumor suppression	[51,62,63,64,66,176,178,260]
MMP-2/9 inhibition	++	++	+	+++	Invasion, metastasis, extracellular matrix remodeling	[67,166,193]
VEGF/angiogenesis suppression	++	+	+	++	Tumor vascularization, metastatic spread	[67,123]
IGF-1/IGF-1R downregulation	+++	+	+	++	Prostate, breast cancer growth, proliferative signaling	[50,51,52,135,176]
DNA methylation/DNMT modulation	++	−	++	+++	Tumor suppressor gene silencing (GSTP1, RARβ2), reversible demethylation	[325,326,327,328,329]
Histone modifications (HAT/HDAC balance)	+	−	++	+++	Chromatin remodeling, RARβ2 reactivation, differentiation	[327,328,329,330]
miRNA regulation	++	−	++	+++	Post-transcriptional control of proliferation, differentiation, apoptosis	[327,331,332,333]

Activity ratings represent the authors’ qualitative assessment of the strength of experimental evidence based on the available literature: +++ strong, ++ moderate, + limited/indirect, − not applicable or not reported. Cx43 = connexin-43; EMT = epithelial–mesenchymal transition; IGF-1 = insulin-like growth factor-1; MMP = matrix metalloproteinase; NF-κB = nuclear factor kappa B; PPARγ = peroxisome proliferator-activated receptor gamma; RAR = retinoic acid receptor; RXR = retinoid X receptor; ROS = reactive oxygen species; STAT3 = signal transducer and activator of transcription 3; VEGF = vascular endothelial growth factor. DNMT = DNA methyltransferase; GSTP1 = glutathione S-transferase pi 1; HAT = histone acetyltransferase; HDAC = histone deacetylase; miRNA = microRNA; RARβ2 = retinoic acid receptor beta2.

**Table 3:** Key Randomized Clinical Trials Evaluating Carotenoids and Retinoids in Cancer Prevention and Treatment.

Trial (Year)	Agent	Population	Dose/Duration	Primary Outcome and Key Finding	Ref
ATBC (1994)	β-Carotene + α-Tocopherol	Male smokers (n = 29,133; Finland)	20 mg/day β-car; 50 mg/day α-toc; 5–8 yr RCT	↑ 18% lung cancer in β-carotene arm; α-tocopherol: no lung benefit but ↓ 32% prostate cancer incidence	[223]
CARET (1996)	β-Carotene + Retinyl palmitate	Smokers and asbestos workers (n = 18,314; USA)	30 mg/day β-car +25,000 IU retinol; ≈4 yr (stopped early)	↑ 28% lung cancer; ↑ 17% overall mortality vs. placebo. Trial halted early due to harm	[222]
PHS I (2000)	β-Carotene	Male physicians (n = 22,071; USA)	50 mg every other day; 12 yr RCT	No significant benefit or harm on overall cancer incidence; no prostate cancer effect	[294]
ATBC—Prostate subanalysis (1998)	β-Carotene + α-Tocopherol	Male smokers (n = 29,133; Finland)	20 mg/day β-car; 50 mg/day α-toc; 5–8 yr	α-Tocopherol: ↓ 32% prostate cancer incidence; β-carotene: no protective effect	[295]
ATBC—Colorectal subanalysis (2000)	β-Carotene + α-Tocopherol	Male smokers (n = 29,133; Finland)	20 mg/day β-car; 50 mg/day α-toc; 5–8 yr	No significant effect of either supplement on colorectal cancer risk in older male smokers	[248]
ATBC—Urinary subanalysis (2000)	β-Carotene + α-Tocopherol	Male smokers (n = 29,133; Finland)	20 mg/day β-car; 50 mg/day α-toc; 5–8 yr	No significant benefit on bladder or renal cancer risk	[299]
CARET— Serum subanalysis (2003)	β-Carotene + Retinyl palmitate	Smokers and asbestos workers (n = 18,314; USA)	Baseline serum micronutrient analysis	Serum carotenoid profiles at baseline did not predict cancer risk modification by supplementation	[291]
EUROSCAN (2000)	Retinyl palmitate ±NAC	Head and neck/ lung cancer patients (n = 2592; Europe)	300,000 IU retinyl palmitate yr 1; 150,000 IU yr 2 ±NAC 600 mg; 2 yr RCT	No benefit on second primary tumors, recurrence, or overall survival; retinoids not recommended as standard chemoprevention in this setting	[321]
Lippman et al. (1993)	Isotretinoin vs. β-Carotene	Oral leukoplakia patients (n = 70)	Isotretinoin 0.5 mg/kg/day vs. β-carotene 30 mg/day; 3 mo + maintenance	Isotretinoin superior for leukoplakia reversal; β-carotene showed minimal activity; long-term remission maintenance challenging	[282]
Nagao et al. (2015)	β-Carotene +Vitamin C	Oral leukoplakia (n = 138; Japan)	β-carotene 30 mg +vitamin C 1000 mg/day; 6 mo RCT	No significant reduction in oral leukoplakia progression to carcinoma	[278]
Mayne et al. (2001)	β-Carotene	Head and neck SCC patients (n = 264; USA)	50 mg/day; 2 yr RCT	No significant reduction in second primary head and neck tumors or recurrence	[280]
Papadimitrakopoulou et al. (2009)	Isotretinoin vs. Retinyl palmitate ±β-Carotene	Oral premalignancy (n = 162)	Isotretinoin 1.5 mg/kg vs. retinyl palmitate ±β-carotene 30 mg; 12 mo	No regimen recommended for oral premalignancy chemoprevention; similar outcomes across all arms	[285]
Veronesi et al. (1999, 2006)	Fenretinide (4-HPR)	Women with early breast cancer (n = 2972; Italy)	200 mg/day; 5 yr RCT; 15-yr follow-up	Significant ↓ second breast cancer in premenopausal women; benefit confirmed at 15-yr follow-up; no overall survival benefit	[322,323]
Duvic et al. (2001)	Bexarotene (RXR-selective retinoid)	Refractory advanced-stage CTCL (n = 94)	300 mg/m^2^/day; Phase II–III trial	Overall response rate 45–55%; FDA-approved for refractory CTCL; first approved rexinoid in oncology	[324]

ATBC = Alpha-Tocopherol, Beta-Carotene Cancer Prevention Study; CARET = Beta-Carotene and Retinol Efficacy Trial; CTCL = cutaneous T-cell lymphoma; EUROSCAN = European Study on Chemoprevention with Vitamin A and N-acetylcysteine; FDA = U.S. Food and Drug Administration; NAC = N-acetylcysteine; PHS = Physicians’ Health Study; RCT = randomized controlled trial; SCC = squamous cell carcinoma; n = total number of participants.

## Data Availability

No new data were created or analyzed in this study. Data sharing is not applicable to this article.
